# A Comparative Study on Delivery of Externally Attached DNA by Papillomavirus VLPs and Pseudoviruses

**DOI:** 10.3390/vaccines9121501

**Published:** 2021-12-18

**Authors:** Sarah Brendle, Nancy Cladel, Karla Balogh, Samina Alam, Neil Christensen, Craig Meyers, Jiafen Hu

**Affiliations:** 1 Jake Gittlen Laboratories for Cancer Research, Pennsylvania State University College of Medicine, Hershey, PA 17033, USA; ganzelly@gmail.com (S.B.); ncladel@gmail.com (N.C.); kkb2@psu.edu (K.B.); ndc1@psu.edu (N.C.); 2 Department of Pathology and Laboratory Medicine, Pennsylvania State University College of Medicine, Hershey, PA 17033, USA; 3 Department of Microbiology and Immunology, Pennsylvania State University College of Medicine, Hershey, PA 17033, USA; sra116@psu.edu (S.A.); cmeyers@pennstatehealth.psu.edu (C.M.)

**Keywords:** virus-like particles (VLPs), L1, L2, pseudovirus (PSV), papillomavirus, DNA delivery, GFP, flow cytometry, HPV16, HPV58, CRPV

## Abstract

Human papillomavirus (HPV) 16 capsids have been chosen as a DNA delivery vehicle in many studies. Our preliminary studies suggest that HPV58 capsids could be better vehicles than HPV16 capsids to deliver encapsidated DNA in vitro and in vivo. In the current study, we compared HPV16, HPV58, and the cottontail rabbit papillomavirus (CRPV) capsids either as L1/L2 VLPs or pseudoviruses (PSVs) to deliver externally attached GFP-expressing DNA. Both rabbit and human cells were used to test whether there was a species-specific effect. DNA delivery efficiency was determined by quantifying either GFP-expressing cell populations or mean fluorescent intensities (MFI) by flow cytometry. Interestingly, CRPV and 58-VLPs and PSVs were significantly more efficient at delivering attached DNA when compared to 16-VLPs and PSVs. A capsid/DNA ratio of 2:1 showed the highest efficiency for delivering external DNA. The PSVs with papillomavirus DNA genomes also showed higher efficiency than those with irrelevant plasmid DNA. HPV16L1/58L2 hybrid VLPs displayed increased efficiency compared to HPV58L1/16L2 VLPs, suggesting that L2 may play a critical role in the delivery of attached DNA. Additionally, we demonstrated that VLPs increased in vivo infectivity of CRPV DNA in rabbits. We conclude that choosing CRPV or 58 capsids to deliver external DNA could improve DNA uptake in in vitro and in vivo models.

## 1. Introduction

Papillomavirus capsids comprise a major capsid protein, L1, and a minor capsid protein, L2 [1,2]. Papillomavirus virus-like particles (VLPs) can be produced from L1 only or L1 and L2 [3]. L1 VLPs have been developed into successful prophylactic vaccines for the control of several human papillomavirus-induced diseases and cancers [4,5]. Papillomavirus L1 VLPs have been shown to bind to several different species of cells and cell types and be internalized by these same cells; therefore [6,7], they are potential vehicles to deliver encapsidated DNA. A previous study suggested that L1/L2 VLPs were more efficient for DNA delivery than L1 VLPs, suggesting that L2 positively impacted the delivery [8]. Pseudoviruses (PSVs, L1/L2 VLPs containing unrelated DNA) have been used to deliver genes, including GFP and luciferase, for in vitro and in vivo studies [9,10,11,12]. Apart from the capability of delivering encapsidated DNA, L1/L2 VLPs have also been shown to efficiently deliver externally attached DNA in vitro and in vivo [13]. This delivery method shows promise because it can deliver a larger amount of DNA when compared with limited copies of encapsidated DNA.

Papillomaviruses do not show a species-specificity in binding and uptake in in vitro cultures [7]. This suggests that different papillomavirus capsids can be used as vehicles for delivering DNA into a variety of cell types. HPV16 was chosen for most delivery studies; however, no studies have compared the delivery efficiency of different types of HPVs. Our unpublished and published studies showed that HPV58 with CRPV DNA genomes was more infectious than HPV16 in vitro and in vivo [14]. Therefore, we hypothesized that different HPV capsids may have diverse inherent efficiencies to also deliver attached DNA to the cells. To test this hypothesis, we designed a series of experiments to compare the delivery efficiency of VLPs (no genome) and PSVs (containing a plasmid or PV genome) made from HPV16 and HPV58 (16-VLP, 58-VLP, PSV-16, and PSV-58) as well as an animal papillomavirus (the cottontail rabbit papillomavirus, CRPV) (CRPV-VLP, PSV-CRPV) in both rabbit and human cell cultures. Our data clearly suggest different delivery efficiencies by several papillomavirus capsid types. Overall, HPV58 capsids were more efficient at delivering both externally attached DNA into rabbit and human cells than HPV16 capsids. CRPV capsids were more efficient for rabbit cells but comparable to HPV58 capsids in human cells. In agreement with previous studies [6,15,16,17,18], we also demonstrated that the L2 protein plays a critical role that contributed to the efficiency of the delivery of attached DNA. Furthermore, we showed that PSVs with a papillomavirus genome showed better delivery of externally attached DNA when compared with PSVs with a plasmid DNA. Our findings suggest that the conformational structure of these particles potentially plays a role in the delivery of attached DNA.

## 2. Material and Methods

### 2.1. L1 VLPs, L1/L2 VLPs, and PSV Production and Validation

**Figure 1:**
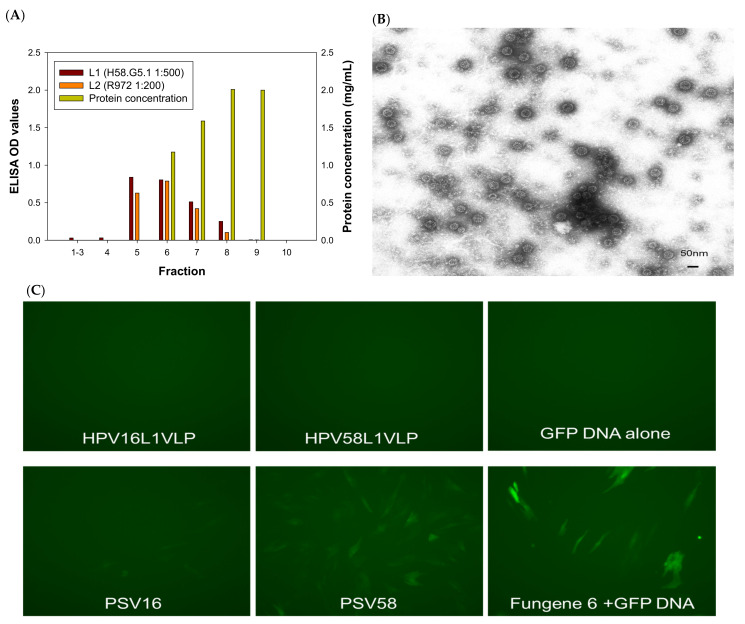

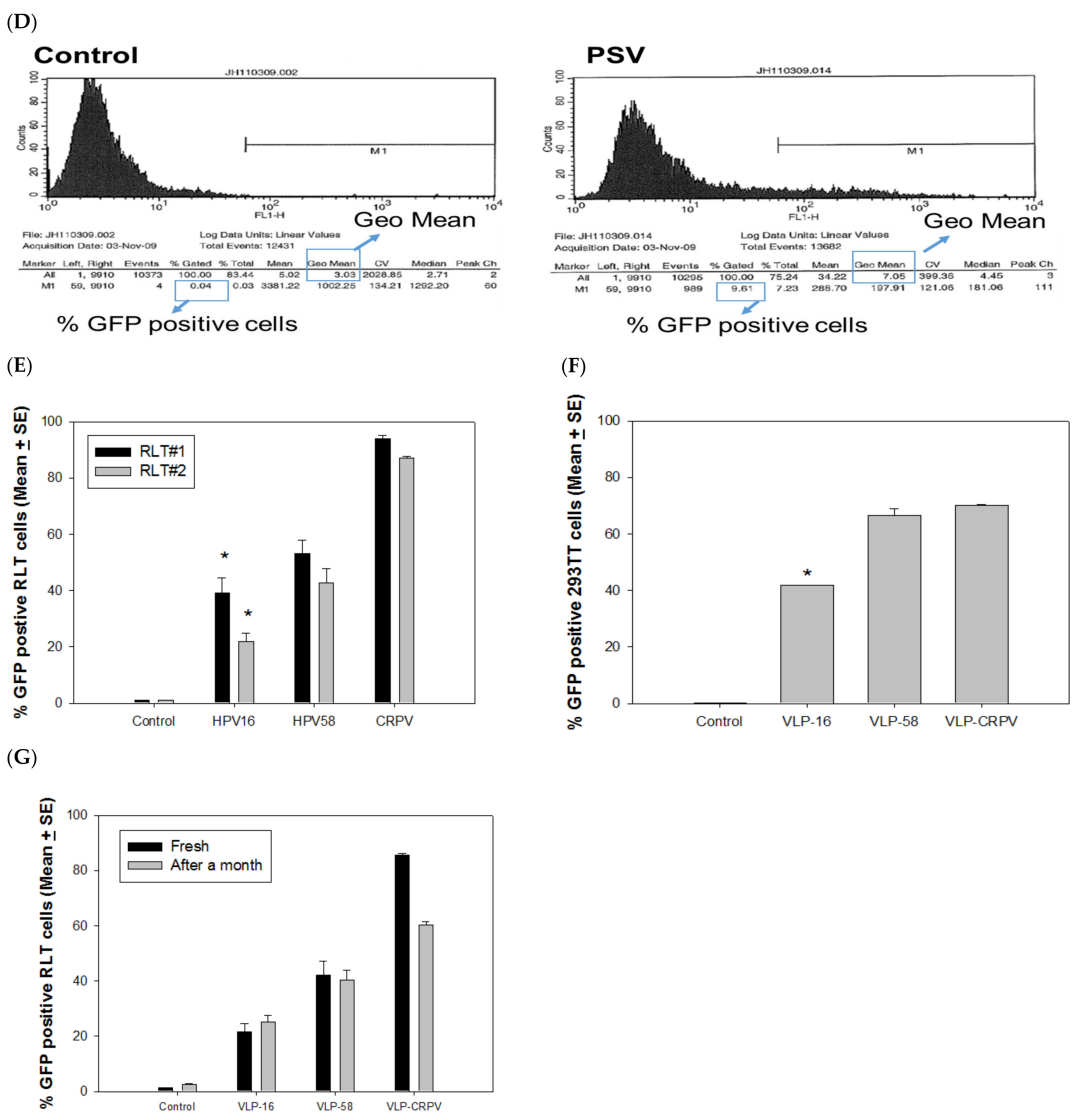
(**A**) Using HPV58 VLPs as an example, L1 and L2 contents were determined by ELISA assay using type-specific antibodies against L1 (H58.G5) and a cross-reacting rabbit antibody against L2 (R972). The corresponding protein concentration in each fraction was also determined by Bio-Rad protein assay (right axis mg/mL). (**B**) VLPs visualized by TEM should be empty particles, but many particles were filled with cellular DNAs and appear full. (**C**) GFP expression in rabbit cells (RLT) by HPV 16 and 58 L1-only VLPs and PSVs. As shown in the top panel, HPV 16 and 58 L1-only VLPs failed to deliver attached GFP into cultured cells. By contrast, GFP-positive cells were found when 16-PSV and 58-PSV delivered attached GFP DNA. 58-PSV showed comparable GFP expression to the commercial transfection kit. (**D**) Flow cytometry analysis was used to quantify GFP signals. Two parameters, the percentage of GFP positive population and mean fluorescent intensity (MFI) by geometric mean were analyzed, as shown here in a typical dataset. These two parameters correlated with each other and thus were used interchangeably for comparison among groups. (**E**) Significantly fewer rabbit cells (cell lines from two different rabbits) were GFP-positive by VLP-16 delivery when compared to HPV58- and CRPV-VLPs (*p* < 0.05, unpaired Mann Whitney test, indicated by *). (**F**) We tested delivery of GFP DNA by HPV58-, 16-, and CRPV-VLPs in 293TT cells. CRPV-VLPs showed comparative efficiency when compared to 58-VLP (*p* > 0.05, unpaired Mann Whitney test). The 16-VLPs showed significantly lower efficacy to deliver DNA when compared with both HPV 58- and CRPV-VLPs (*p* < 0.05, unpaired Mann Whitney test, indicated by *). (**G**) To determine the stability of VLPs and PSVs, we tested fresh VLPs with those stored at −20 °C for a month. We observed that CRPV-VLP delivery efficiency decreased, while HPV16- and 58-VLPs maintained the same level of potency for delivery.

L1, L1/L2 VLPs, and pseudoviruses (PSVs) were produced in 293TT cells as reported previously with some modifications (Table 1) [19]. In brief, 293TT cells maintained in DMEM complemented with hygromycin were used for transfection. No antibodies were added to the medium during DNA transfection using Lipofectamine 2000 (Invitrogen). The transfected cells were split 1:2 and cultured in a medium with 50 units/mL penicillin/streptomycin. Transfected cells were harvested at 48 h post-transfection and allowed to mature at 37 °C overnight. DNA contamination was eliminated by adding Benzonase (Sigma) and plasmid-safe exonuclease (NEB) during OptiPrep gradient ultracentrifugation. A panel of plasmid DNA with different sizes, including the PCX expression vector (5.5 kb), the secreted alkaline phosphatase (5.5 kb, SEAP), and the CRPV genomic DNA (7.8 kb), were used for PSV production (23–25). All plasmid DNA was prepared by the QIAGEN maxiprep kit. For PSVs, 20 µg of plasmid DNA was co-transfected with L1 and L2 DNA of the relevant HPV type (HPV16 and HPV58) or CRPV. Hybrid HPV16L1/58L2, HPV58L1/16L2 VLPs, and corresponding PSVs were also produced. VLPs were produced by transfecting L1 or L1/L2 plasmids without any genomic DNA plasmids. All VLPs and PSVs were purified by OptiPrep ultracentrifugation and different fractions were examined by ELISA for both L1 (in house monoclonal antibodies, H16.V5, H58.G5.1, and CRPV.A4 were used to detect HPV16, HPV58, and CRPV L1, respectively) and L2 (rabbit polyclonal antibody R972 responding to the conserved region of HPV L2) (Table 1). Figure 1A shows representative HPV58 VLP L1 and L2 detection by ELISA. The fractions from VLPs and PSVs positive for both L1 and L2 were pooled (fraction #5–7 in this example) as the stock for experiments in the current study. The protein concentration for each preparation was determined by Bio-Rad protein analysis using different concentrations of BSA as the standard curve (Figure 1A). We prepared several batches of VLPs and PSVs for repeated experiments. VLP particles were also examined by TEM using negative staining, as reported previously (Figure 1B) [20]. All preparations were stored in OptiPrep at 4 °C for short-term storage or −20 °C for long-term storage.

### 2.2. Cells for Externally Attached DNA Delivery

Rabbit cells (primary cultures from two individual rabbits or SV40LT immortalized cell lines based on the primary cultures designated as RLT, and an inbred rabbit cancer cell line, I3) and human cell lines (293TT cells and SiHa cells) were maintained in DMEM. Mouse dendritic cells (mDCs, cultured from previously harvested mouse bone marrow cells as described previously [21,22]) were maintained in RPMI1640. Both media were supplemented with 10% FBS, 50 units/mL penicillin/streptomycin, 2 mM glutamine, and 1 mM Na pyruvate. Cells were cultured at 37 °C with 5% CO_2_. All cell lines were seeded at a density of 3 × 10^5^/well into 24-well plates and tested after reaching 70–80% confluence.

### 2.3. External DNA Delivery Using VLPs and PSVs in In Vitro Cell Cultures

Green fluorescent protein (GFP) DNA was cloned into a mammalian expression vector PCR3 (Invitrogen) and confirmed by DNA sequencing at the Core Facility of Penn State College of Medicine. Different amounts of VLPs or PSVs were incubated with different amounts of GFP DNA at room temperature for 5 min for the delivery of attached DNA. Triplicate wells in a 24-well plate were used for each condition. The mixtures were then added to the cells and cultured for 48–72 h. The cells were subsequently harvested and fixed in 1 × PBS with 2% paraformaldehyde for flow cytometry using one-color (FITC) analysis.

### 2.4. Fluorescent Detection and Quantification of Delivery Efficacy Using Flow Cytometry Analysis

The GFP-positive cells were visualized under a fluorescent microscope, and a representative image is shown in Figure 1C at 48–72 h post-transfection. The cells were also collected and fixed with 2% paraformaldehyde for further analysis by flow cytometry at the Core Facility of Pennsylvania State University College of Medicine. To quantify the efficacy of DNA delivery, we measured two parameters: the percentage of GFP positive population and mean fluorescent intensity (MFI) using the geometric mean. An example dataset is shown (Figure 1D). These two parameters correlated with each other and thus were used interchangeably for our comparison among groups in the following experiments. FlowJo software was used for single-color data analysis.

### 2.5. VLP Delivery of Viral DNA for In Vivo Infection of Rabbits

All work on rabbits was approved by the Institutional Animal Care and Use Committee of the Pennsylvania State University College of Medicine (PSUCOM), and all procedures were performed in strict accordance with guidelines and regulations. Two New Zealand White Rabbits were used to test the delivery of DNA with CRPV-VLPs in vivo. We used several low doses (10, 20, and 40 ng) of CRPV DNA alone or DNA mixed with 20, 40, and 80 ng of CRPV-VLPs and delivered the DNA onto the pre-wounded back skin sites of rabbits as described previously [23]. Tumor outgrowth was monitored and recorded in millimeters as length × width × height. A geometric mean was calculated for each tumor. The tumor appearance (tumor sites/infected sites) of each combination was recorded.

### 2.6. Statistics

Mean fluorescent intensity (MFI) and the geo mean and percentage (%) of GFP positive cells were compared among different groups for each experiment according to types of VLPs and PSVs. Data were entered into SigmaPlot, and means and standard errors (SEM) for each test group were calculated. Plots were generated using SigmaPlot. Unpaired Mann Whitney tests were used to identify the significance (*p* < 0.05 was considered a significant difference between samples).

## 3. Results

### 3.1. L2 Is Required for Delivering Externally Attached DNA to the Cells In Vitro

Previous studies demonstrated that HPV16 L1 VLPs failed to carry attached DNA into human cells [13]. To investigate whether this was due to a papillomavirus type-specific characteristic, we tested 16- and 58-L1 VLPs by mixing the corresponding L1 VLPs with a GFP-expressing DNA and incubating them at room temperature for 5 min before adding the mixtures to RLT cells [12,24,25]. GFP expression in RLT cells was examined by fluorescence microscopy 48 h later. Consistent with previous findings for 16 L1-VLPs, no GFP expression was found from any of the L1 VLPs tested (Figure 1C). By contrast, PSV16 and PSV58 showed GFP-positive populations. PSV58 showed similar GFP expression when compared to GFP DNA transfection by the transfection reagent FuGENE 6. More GFP-positive cells were found in the PSV58 and FuGENE 6 control group when compared with the PSV16 group (Figure 1C, bottom panel). To quantify the GFP-positive cells, we harvested cells for flow cytometry, as shown in Figure 1D. We used both the percentage of GFP-positive (an arbitrary measure set for all samples in one test) and the Geo Mean for the mean fluorescent intensity (MFI) to compare groups. These two parameters correlated with each other, and both were used in the analysis interchangeably. All capsids used in subsequent studies contained L2, and thus 16-VLP indicates L1/L2 VLPs and all delivery tests were externally attached GFP DNA.

### 3.2. PSVs and VLPs Deliver Attached DNA to Both Rabbit and Human Cells

The N-terminus of HPV16 L2 interacts with HPV16 DNA [7], and the N-terminus of BPV-1 L2 also displays on the surface of the BPV-1 capsid [16]. These observations suggest that the N-terminus of L2 on the capsid surface could interact with attached DNA, leading to DNA delivery to cells [13]. Our preliminary study demonstrated that HPV58 capsids containing the cottontail rabbit papillomavirus (CRPV) DNA genome showed higher infectivity when compared with corresponding HPV16 capsids (unpublished observations). We hypothesize that 58-VLPs may be more efficient than 16-VLPs to deliver attached DNA in vitro and in vivo.

To compare the delivery efficiency of different capsids, we incubated 1 µg of 16-, 58- and CRPV-VLPs with 0.5 µg of GFP-expressing DNA. The mixtures were then added to two SV40 LT immortalized rabbit cell lines (RLT#1 and RLT#2). All three VLP types successfully delivered GFP DNA into the rabbit cells as determined by a GF- positive population (Figure 1E). Interestingly, CRPV-VLPs showed the highest delivery efficiency, and a significant difference was also found between 58- and 16-VLPs, with 16-VLPs delivering the least amount of DNA (Figure 1E, *p* < 0.05, unpaired Mann Whitney test). Therefore, 58-VLPs are more efficient than 16-VLPs for the delivery of attached DNA. The results suggest that VLPs could be used as vectors to deliver attached DNA to cells efficiently and that the delivery efficiency is papillomavirus type-dependent.

Our studies described above were performed using rabbit cells, and therefore, we questioned whether these different VLPs displayed a similar pattern in human cells. We used 293TT cells to test delivery by adding GFP DNA attached to 58-, 16-, and CRPV-VLPs (the same batch of VLPs we used for the above experiment, as shown in Figure 1E). Three days after, the cells were tested for GFP expression. CRPV-VLPs showed comparable efficiency with 58-VLPs in 293TT cells (Figure 1F). In both rabbit and 293TT cells, 16-VLPs were the least efficient for DNA delivery (Figure 1E,F, *p* < 0.05, unpaired Mann Whitney test). Therefore, 58-VLPs are superior to 16-VLPs for the delivery of attached DNA in both human and rabbit cells.

All particles were stored in OptiPrep at 4 °C for short-term storage or −20 °C for long-term storage. We further tested the stability of these VLPs after storage for a month at −20 °C. We conducted the delivery experiment in the same rabbit cell line in which we tested the fresh VLP stocks above. As shown in Figure 1G, CRPV-VLPs showed slightly decreased delivery efficiency, while both 16- and 58-VLPs maintained the same potency.

### 3.3. PSVs Were More Efficient in Delivering DNA When Encapisidating a Papillomavirus Genome DNA

**Figure 2:**
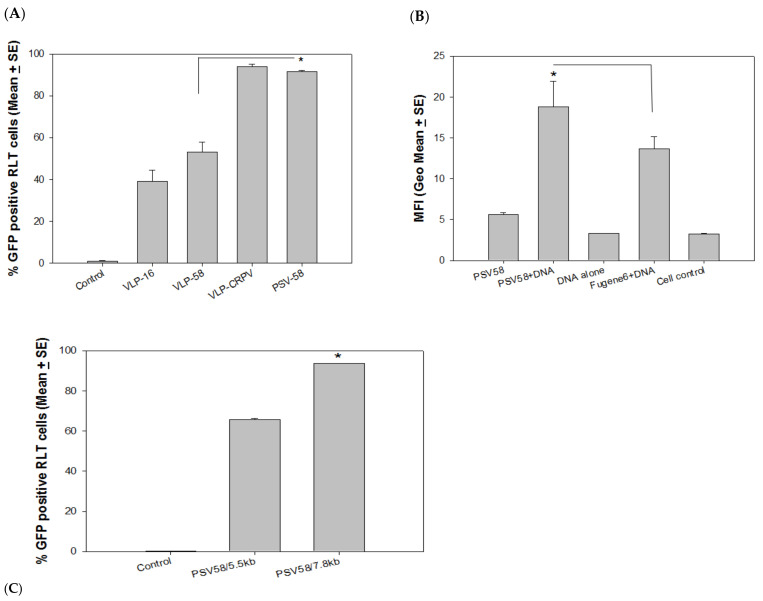
(**A**) PSV58 encapsidating an unrelated 5.5 kb DNA plasmid (PSV58/5.5 kb) showed improved delivery over VLP-58 (*p* < 0.05, unpaired Mann Whitney test, indicated by *) that was also comparable to VLP-CRPV. (**B**) External GFP DNA delivered by PSV58 was more efficient than a commercial transfection reagent (*p* < 0.05, unpaired Mann Whitney test, indicated by *). No difference was found between PSV58 alone, GFP DNA alone, and medium control (*p* > 0.05, unpaired Mann Whitney test). (**C**) Efficiency of external DNA delivery is significantly higher by PSV58 containing the CRPV genome when compared to PSV58/5.5 kb (PCX expression vector, 5.5 kb) genome (*p* < 0.05, unpaired Mann Whitney test, indicated by *).

PSV58 encapsidating an unrelated PCX plasmid (5.5 kb) (designated as PSV58/5.5 kb) showed improved delivery of attached DNA when compared to 58-VLPs (Figure 2A, *p* < 0.05, unpaired Mann Whitney test) in the rabbit cells. The signal was comparable with CRPV-VLPs. This suggests that PSV58 was more efficient than corresponding VLPs for delivering external DNA. The experiment was repeated to compare PSV58 with the commercial transfection reagent FuGENE 6. We detected significantly higher MFI in the group delivered by PSV58 when compared to those transfected by FuGENE 6 (Figure 2B, *p* < 0.05, unpaired Mann Whitney test). This finding suggests that PSV58 could be used as a vehicle for DNA transfection in vitro.

Papillomavirus genomes are preferred to be packed inside papillomavirus capsids in vitro [26,27]. To determine whether including a papillomavirus genome would further improve the delivery efficiency of attached DNA, we produced PSVs encapsidating the CRPV genome (identified as PSV58/7.8 kb) to compare with PSV58/5.5 kb in rabbit cell cultures. Significantly more GFP-expressing cells were found to be delivered by PSV58/7.8 kb when compared with those delivered by PSV58/5.5 kb (Figure 2C, *p* < 0.05, unpaired Mann Whitney test). Therefore, PSVs with a papillomavirus genome DNA appear to deliver attached DNA more efficiently than particles encapsidating a plasmid DNA (5.5 kb). We also tested a panel of PSV58 encapsidating different sizes of plasmid DNA but did not observe increased delivery of external DNA with an increase in DNA size (data not shown). This may indicate that PSVs containing the papillomavirus genome improve external DNA binding and delivery, as the size of encapsidated DNA alone is not necessarily correlated with improved delivery.

## 4. Optimal VLP/External DNA Ratio for Delivery

**Figure 3:**
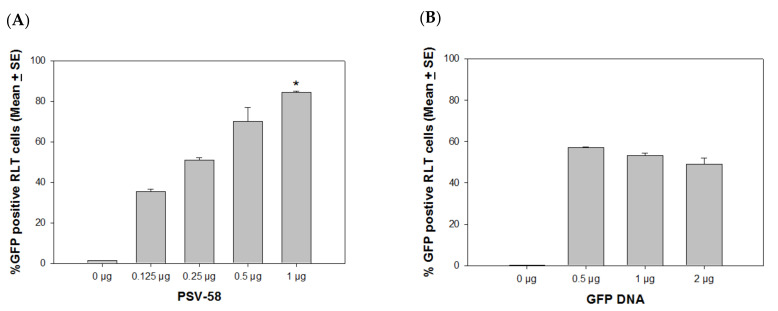
Dose curve to determine the optimal capsid/DNA ratio. (**A**) Different amounts of PSV58 (0.125, 0.25, 0.5, and 1 µg) were mixed with 0.5 µg of GFP DNA and added to SV40 LT immortalized rabbit cells (RLT). The 1 µg PSV-58 group showed increased numbers of positive cells when compared with the 0.125 and 0.25 µg groups (*p* < 0.05, unpaired Mann Whitney test, indicated by *) (**B**) A range of GFP DNA was delivered by 1 µg of PSV58 to test for optimum delivery. An increased amount of GFP DNA did not show increased delivery efficiency; 1 µg PSV58 delivering 0.5 µg GFP DNA was the most efficient ratio for external DNA delivery.

Since we observed that VLPs and PSVs are promising delivery vehicles for external DNA, we sought to determine the optimal ratio between capsid and GFP DNA. For this experiment, we chose PSV58 to optimize the conditions, as this PSV58 was more efficient than PSV16 for external DNA delivery. We first determined the highest amount of PSV needed for the delivery of 0.5 µg GFP DNA and identified that 1 µg PSV58 achieved close to saturation levels of expression in a 24-well plate (Figure 3A, *p* < 0.05, unpaired Mann Whitney test). Next, we mixed 1 µg of PSV58 with 0, 0.5, 1, or 2 µg of GFP DNA (a ratio of 2:1, 1:1, or 1:2) and tested the different combinations in RLT cultures. An increased amount of GFP DNA did not show increased delivery efficiency (Figure 3B, *p* > 0.05, unpaired Mann Whitney test). The capsid/DNA ratio of 2:1 induced the strongest GFP expression when compared with the other ratios. The following experiments were conducted with the optimal 2:1 VLPs/DNA ratio.

### 4.1. PSVs Deliver DNA More Efficiently in Immortalized Cells vs. Normal Cells

**Figure 4:**
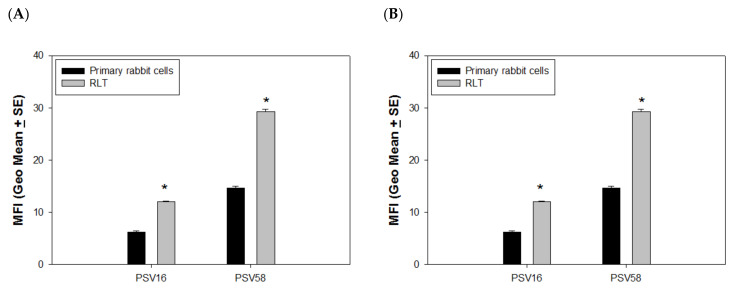
Delivery efficacy was determined by cell status. (**A**) Higher delivery efficacy was found in immortalized cells when compared to primary cells (*p* < 0.05, unpaired Mann Whitney test, indicated by *). (**B**) Significantly higher delivery efficacy was found in PSV58 when compared with PSV16 (*p* < 0.05, unpaired Mann Whitney test, indicated by *) in mouse dendritic cells.

VLPs delivering external DNA could be a promising future gene therapy for the treatment of different diseases. To examine whether the capsids have advantages in delivering GFP DNA to immortalized cells compared to primary cells, we compared the efficiency of PSV16, PSV58, and PSVCRPV to deliver external GFP DNA to both primary and immortalized rabbit cells with the same method described above. Again, PSV58 was more efficient in delivering GFP DNA in both normal and transformed cells when compared with PSV16. Most interestingly, we observed that significantly higher levels of GFP were found in immortalized cells (RLT) when compared with primary cells for both PSV58 and PSV16 (Figure 4A, *p* < 0.05, unpaired Mann Whitney test), and again, PSV58 delivered more GFP when compared with PSV16. We also examined whether these PSVs could deliver external DNA to antigen-presenting cells and used cultured mouse dendritic cells for this experiment. Although the delivery efficiency of all VLPs was much lower in DCs when compared with RLT or 293TT cells, we did find a similar pattern as seen in the primary rabbit cells in that PSV58 was more efficient than PSV16 (Figure 4B, *p* < 0.05, unpaired Mann Whitney test).

### 4.2. L2 Played a Critical Role in Delivery Efficiency

**Figure 5:**
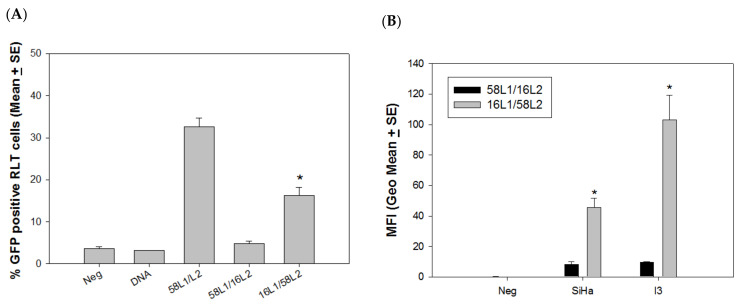
Delivery efficacy of external DNA is L2-dependent. Two hybrid VLPs, 16L1/58L2 and 58L1/16L2, were tested for attached DNA delivery with GFP DNA in rabbit and human cells. (**A**) The delivery efficiency of hybrid VLP-16L1/58L2 was partially restored in RLT cells when compared with VLP-58L1/16L2. Significantly more cells expressed GFP when delivered by VLP-16L1/58L2 (similar to VLP-58) when compared to VLP-58L1/16L2 (similar to VLP-16) in rabbit cells (*p* < 0.05, unpaired Mann Whitney test, indicated by *). (**B**) Additionally, two more cell cultures (a rabbit cell line (I3) and a human cell line (SiHa)) were tested for hybrid VLP delivery. A similar pattern was found, as shown in RLT cells in the above experiments (*p* < 0.05, unpaired Mann Whitney test, indicated by *).

The previous experiments demonstrated that L1-only VLPs failed to deliver external DNA, but VLP-58 was advantageous when compared to VLP-16 in delivering external DNA in both rabbit and human cells. Therefore, we hypothesized that L2 played a critical role in this delivery method. To test this hypothesis, we produced two hybrid VLPs (HPV58L1/16L2 and HPV16L1/58L2). These two hybrid VLPs were tested for the delivery of external GFP DNA to RLT cells. Hybrid VLP-16L1/58L2 showed significantly higher delivery efficiency when compared with VLP-58L1/16L2 (Figure 5A, *p* < 0.05, unpaired Mann Whitney test), although the delivery of both was significantly lower than that of VLP-58. We also tested these hybrid VLPs in two immortalized cell lines, including rabbit (I3) and human (SiHa) cell lines. Consistent with what is shown in Figure 5A, hybrid VLP-16L1/58L2 showed significantly higher delivery efficiency when compared with VLP-58L1/16L2 (Figure 5B, *p* < 0.05, unpaired Mann Whitney test) in both human and rabbit cells. Our results suggest that the L1/L2 VLP delivery efficiency is potentially an L2-dependent phenomenon.

### 4.3. VLP-CRPV Increased Viral Infectivity In Vivo

To further determine whether VLP-CRPV could deliver DNA in vivo, we tested different low doses of CRPV DNA with and without VLPs for delivery and infectivity in a rabbit model. VLP-CRPV was mixed in a 2:1 ratio with 10, 20, and 40 ng of CRPV DNA and infected at two pre-wounded back skin sites of New Zealand White Rabbits as described previously [23]. The rabbits were monitored for tumor growth and recorded by photography. As shown in Table 2, more tumors were observed in groups where the DNA was delivered by VLPs compared to the DNA alone group. This effect was greater in the groups with a lower dose of DNA. However, the difference in the groups was not significant because of the small sample size (*p* > 0.05, unpaired Mann Whitney test).

## 5. Discussion

Previous studies have demonstrated that HPV capsids assembled as pseudoviruses can effectively deliver encapsidated DNA into cells and are considered promising vehicles for gene therapy and DNA vaccination because HPV VLPs and PSVs can be taken up by different cell types [7,28,29,30]. More recently, L1/L2 VLPs but not L1 VLPs have been found to effectively deliver externally attached DNA in vitro and in vivo [13]. HPV16 capsids were used for most DNA delivery studies. Our previous unpublished and published studies suggested that HPV58 L1/L2 encapsidating CRPV DNA was more infectious than HPV16 L1/L2 encapsidating CRPV DNA [14]. In the current study, we tested our hypothesis that different HPVs may have diverse inherent efficiencies to deliver attached DNA to the cells using VLPs or PSVs (containing a plasmid DNA). We tested HPV16, HPV58, and CRPV and a variety of cell lines, including rabbit, human, and mouse dendritic cells. Consistent with previous findings, L1-only VLPs from all PV types failed to deliver attached DNA into cells, indicating that L1 alone is not sufficient. By contrast, all L1/L2 VLPs and PSVs tested successfully delivered externally attached DNA into both rabbit and human cells despite the difference in delivery efficiencies. VLP-58 showed significantly higher delivery efficacy when compared to VLP-16. Interestingly, VLP-CRPV showed higher delivery to rabbit cells, but the delivery was comparable to 58-VLPs in human cells. In addition, the delivery efficacy of attached DNA was L2-dependent because hybrid VLP-16L1/58L2 showed increased efficacy when compared to hybrid VLP-58 L1/16L2 in both rabbit and human cells.

Both L1 and L2 contain DNA binding sites [6,7,15,31]. At the C-terminus, nine amino acids have been demonstrated essential for DNA binding and gene delivery by L1 VLPs [32]. Unrelated DNA can be encapsidated into L1-only particles by reassembly in vitro and can thus be delivered into cells [33,34]. The efficacy of this delivery has been characterized in vitro and in vivo [5,28,35,36]. Although L1 can successfully encapsidate DNA and form infectious PSVs, it failed to bind external DNA in previous studies and in our studies, indicating that the DNA binding sites of L1 might be embedded inside the particles [13]. The L2 protein has been found to be critical for the infectivity of PSVs [37]. Several DNA binding sites have been identified in HPV L2. Three conserved regions that are important for neutralizing and cross-neutralizing antibody targets have been identified in L2: (1) an N-terminal external loop of L2 (L2/17-36aa) has been identified as the target of neutralizing and cross-neutralizing antibodies [18,38]; (2) an N-terminal region (L2/108-120aa) has been identified as a surface epitope that shares a common neutralizing epitope for HPVs [37,39,40]; and (3) a C-terminal region of L2 has a lysine–arginine-rich sequence (RKRRKR in HPV16 L2). This latter region is also identified as an important L1-L2 interaction region [6,41]. Higher efficacy in delivering externally attached DNA by HPV58 L2 compared to HPV16 L2 implicates that one of these conserved regions of L2 might play a critical role [42]. A 23 amino acid membrane-destabilizing L2 peptide located at the C-terminus of L2 has been found to be critical for external DNA binding [8,13]. It would be interesting to identify the critical DNA binding sites in this region for HPV58. We found that the L1 and L2 interaction between hybrid HPV16 and HPV58 L1/L2 VLPs was not interrupted in terms of capsid formation and encapsidating DNA. The compatibility of L1 and L2 between HPV16 and HPV18 in encapsidating DNA genomes has been reported previously [43], as these capsids contain highly conserved regions among the HPV L2 proteins. We postulate that L2, rather than the interaction between L1 and L2, may play a predominant role in this externally attached DNA delivery because the two hybrid VLPs HPV16/HPV58 were both capable of delivering DNA to cells. The identification of the nucleus-guided sequences from L2 would also be valuable for the design of future DNA carriers for gene therapy.

The difference in DNA binding between VLPs and PSVs has not been studied extensively. In this study, we demonstrated PSVs displayed higher delivery of attached DNA when compared with corresponding VLPs, while PSVs containing papillomavirus DNA were superior to PSVs containing 5.5 kb DNA in delivering attached DNA. This suggests a possible conformation structure difference among particle types, which may play a role in the external binding of DNA. In the current study, we know both VLPs and PSVs could potentially contain cellular DNA from production in the 293TT culture system. However, we argue that PSVs have more particles containing the target DNA, leading to a more rigid structure when compared with the less “filled” capsids (VLPs), and this may contribute to the higher binding/delivery [20]. How different sizes of encapsidated DNA influences the delivery efficacy of attached DNA needs further investigation.

Another interesting finding is that much higher DNA delivery was detected by externally attached DNA vs. the delivery of genome DNA encapsidating as PSVs (data not shown). Our unpublished study showed that only one copy of full-length DNA can be encapsidated because of the restriction of the space inside the capsid. For externally attached DNA delivery, excess DNA was incubated with the VLPs or PSVs, and therefore, significantly higher amounts of DNA could be attached to the particles. The mechanism by which attached DNA is carried into the cells without being damaged by nucleases is unclear. Our recently published study demonstrated that the papillomavirus genome could be delivered intravenously into the circulatory system and generate local lesions in the rabbit model [44]. We hypothesize that VLPs and PSVs may have provided some protection for externally attached DNA from nuclease digestion.

A previous study showed that a ratio of 1:5 between VLP-16: GFP DNA attained the highest level of GFP expression in animals 7 days after intradermal injection [13]. In our in vitro culture studies, we tested different doses of GFP DNA and identified a saturating dose that led to high expression in RLT cells. We further tested VLP-58 and GFP DNA for optimal delivery and demonstrated that a ratio of 2:1 was optimal for in vitro delivery. We used this ratio for the VLP-CRPV/CRPV genome for in vivo delivery. Whether the difference in our findings vs. those of the previous study resulted from the differences between in vitro (cell types, the length of incubation time, etc.) and in vivo systems (gene delivery vs. infection-induced tumor growth) needs further investigation.

## 6. Conclusions

In conclusion, our study demonstrated that HPV58 and CRPV capsids are advantageous to HPV16 for delivering attached DNA, and the delivery efficacy is L2-dependent. This finding agrees with our previous observation that the HPV58 capsid delivered attached DNA more efficiently than HPV16. The HPV L1/L2 VLPs/externally attached DNA method can be applied to both in vitro transfection studies (because comparative transfection efficiencies were found between VLPs and the commercial transfection reagents) and in vivo viral DNA infection, as higher and more consistent infectivity could be achieved. Our findings further demonstrated that the delivery of attached DNA by HPV VLPs was not species-specific, and delivery was preferential in immortalized cells compared to normal cells. Chimeric HPV VLPs have been tested for their function in activating antigen-presenting cells, such as Langerhans and dendritic cells, in vitro and in vivo [13,45,46,47,48,49]. These characteristics can be potentially used for therapeutic DNA vaccination and tumor treatment using different HPV capsids.

## Figures and Tables

**Table 1:** Viral particles (VLPs) and pseudoviruses (PSVs) used in the current study.

Name	L1	L2	DNA Plasmid	Confirmation by ELISA	Notes
L1 VLPs	Yes	No	No	Antibodies used for **L1**: H16.V5 for HPV16L1, H58.G5 for H58L1, and CRPV.A4 for CRPV L1 **L2**: (R972 1:100)	All VLPs and PSVs were produced in 293TT cell culture, purified using OptiPrep gradient ultracentrifugation, and stored at −20 °C. For VLPs and PSVs, the positive L1 and L2 fractions were pooled, and concentrations were determined by Bio-Rad protein assay
VLPs (L1/L2)	Yes	Yes	No		
PSVs	Yes	Yes	Yes, (pSEAP, 5.5 kb, PCX 5.5 kb, and the CRPV genome 7.8 kb)		

**Table 2:** CRPVL1/L2 VLPs increase the infectivity of DNA infection in vivo.

Dose (ng)		Tumor Outgrowth (Tumor Sites/Challenge Sites)	Average Tumor Size (Geo Mean in mm)
DNA	VLPs		
10	-	1/4 (25%)	0.5000
10	20	1/2 (50%)	3.0350
20	-	2/4 (50%)	3.8675
20	40	2/2 (100%)	7.8550
40	-	3/4 (75%)	6.3750
40	80	2/2 (100%)	7.2500

## Data Availability

Not applicable.
